# Perinatal factors associated with early neonatal deaths in very low birth weight preterm infants in Northeast Brazil

**DOI:** 10.1186/s12887-014-0312-5

**Published:** 2014-12-20

**Authors:** Eveline Campos Monteiro de Castro, Álvaro Jorge Madeiro Leite, Maria Fernanda Branco de Almeida, Ruth Guinsburg

**Affiliations:** Neonatal Unit of Maternidade Escola Assis Chateaubriand, Universidade Federal do Ceará, 3678 aptº 1600 – Meireles, CEP: 60165-121 Fortaleza, CE Brazil; Department of Maternal and Child Health, Universidade Federal do Ceará, Fortaleza, Ceará Brazil; Department of Pediatrics, Escola Paulista de Medicina, Universidade Federal de São Paulo, São Paulo, São Paulo Brazil

**Keywords:** Premature newborn infant, Very low weight newborn infant, Neonatal mortality, Early neonatal mortality, Neonatal ICU

## Abstract

**Background:**

In Brazil, the prevalence of prematurity has increased in recent years and it is a major cause of death in the neonatal period. Therefore, this study aims at assessing perinatal factors associated with early neonatal deaths in very low birth weight preterm infants born in a region of Brazil with low Human Development Index.

**Methods:**

Prospective cohort study of inborns with gestational age 23^0/7^-31^6/7^ weeks and birthweight 500-1499 g without malformations in 19 public reference hospitals of the state capitals of Brazil’s Northeast Region. Perinatal variables associated with early neonatal death were determined by Cox regression analysis.

**Result:**

Among 627 neonates, 179 (29%) died with 0–6 days after birth. Early death was associated to: absence of antenatal steroids (HR 1.59; 95% CI 1.11-2.27), multiple gestation (1.95; 1.28-3.00), male sex (2.01; 1.40-2.86), 5th minute Apgar <7 (2.93; 2.03-4.21), birthweight <1000 g (2.58; 1.70-3.88), gestational age <28 weeks (2.07; 1.42-3.02), use of surfactant (1.65; 1.04-2.59), and non-use of a pain scale (1.89; 1.24-2.89).

**Conclusion:**

Biological variables and factors related to the quality of perinatal care were associated with the high chance of early death of preterm infants born in reference hospitals of Northeast Brazil.

## Background

Neonatal mortality has become increasingly the most important component of infant mortality. The slow reduction of neonatal mortality rate in poor or developing countries is worthy of attention. Of all neonatal deaths, three quarters occur in the first week of life [1].

In Brazil, the neonatal mortality rate remains high, at 10 out of every 1,000 live births in 2011, and accounts for 70% of infant mortality. Post-neonatal components of infant mortality were largely reduced throughout the country due to improvements in primary health care, but neonatal deaths in the first week of life have increased from 50% of infant deaths in 2000 to 53% in 2010, and 26% of these deaths occur on the first day of life [2]. In the Northeast region of Brail, the early neonatal mortality rate (11.6/1,000 live births) is twice as high as that of the South (5.9/1,000 live births). Mortality during the first day of life is becoming an increasingly large contributor to the overall infant mortality rate in the Northeast, rising from 23% in 2000 to 28% in 2010, while the opposite trend was observed in the Southeast, where mortality in the first day of life was reduced from 27% in 2000 to 24% in 2010 [2].

In Brazil, the prevalence of prematurity has increased in recent years due to poor quality of reproductive and prenatal health care and the misuse of medical interventions during childbirth [3,4]. This increase is a concern because prematurity remains a major cause of death in the neonatal period [4-6].

Given this background, the present study sought to evaluate the factors associated with the early neonatal deaths of very low birth weight (VLBW) preterm infants born in public hospitals in the state capital cities of northeastern Brazil.

## Methods

This study is a retrospective analysis of a prospectively obtained regional database that included live births with gestational ages between 23^0/7^ and 31^6/7^ weeks, weighing between 500 and 1499 g, born in 19 public reference maternity units in the capitals of the nine Northeastern states in the period between July and December of 2007. Patients with major congenital malformations, those transferred from other institutions and those who died in the delivery room were excluded. The study used the database of the North-Northeast Perinatal Health Network (Rede Norte-Nordeste de Saúde Perinatal - RENOSPE), which was an initiative of the Ministry of Health. The project was approved by the Clinical Directors of all participating hospitals and by the Ethical Research Committees of the Federal University of Ceará and of the Federal University of São Paulo. The Clinical Board of each participating institution approved the study protocol.

The research developed by RENOSPE, using data collected from neonatal intensive care units (NICUs), evaluated 36 hospitals in the Northeastern states. RENOSPE database is not publicly available, but access to data can be obtained by contact with one of the authors (AJML). The present study examined 29 hospitals located in the state capitals. Two of these hospitals were excluded because they lacked maternity units and eight others were excluded because they did not report all patients born during the collection period. Therefore, 19 public hospitals were included from nine Northeastern capitals. The total number of beds in the NICUs was 236, ranging from six to 21 beds per unit, with a median of 10 beds per unit.

The 19 hospitals were evaluated using a questionnaire that assessed the physical facilities, equipment, human resources and quality care initiatives. The questionnaire was completed by managers and health professionals. To categorize the neonatal units, the above characteristics were weighted so that the features present in most hospitals had lower scores than those present in a minority of hospitals; i.e., the greater the number of hospitals with a certain characteristic, the lower the weighting in the hospital level classification and vice versa. Two categories were proposed based on this score: Level 1 (L1) for those hospitals with a better infrastructure (score: 61-100%) and Level 2 (L2), for those with a less equipped infrastructure (score: 35-60%).

Data collection in each unit, from the time of admission until discharge or death, was carried out prospectively from the medical records of the mother and newborn by a field researcher (doctor or nurse) trained by RENOSPE coordinators. Data collection included maternal and neonatal demographic characteristics, neonatal morbidity and variables related to procedures and interventions in neonatal care. The evaluation of pain at any point during hospitalization was defined as the use of any validated pain scale for the newborn. The outcome variable was death in the first 0–6 days after birth.

The probability of newborn survival was calculated using the Kaplan-Meier method. A Cox regression model was fitted to verify the associations of the independent factors with the outcome of early neonatal death. The behavior of each independent variable (hospital category, maternal and neonatal characteristics, clinical complications and the use of procedures and interventions in the first week of life) was evaluated using Kaplan-Meier and compared by the log-rank test. All variables with p <0.20 in this analysis were included in the initial Cox regression model and then removed one by one if p <0.05. The Cox regression model associations were expressed with a *hazard ratio* (HR) and its 95% confidence interval (95% CI). SPSS 17.0 software was used for all statistical analyses, with a significance level of p <0.05.

## Results

Between July and December 2007, a total of 27,991 live births occurred in the 19 public reference hospitals in the capitals of the Northeast region included in the study. Of these, 1,010 newborns weighing 500–1499 g were admitted to neonatal units (4% of births) and 383 were excluded: 75 with congenital malformations, 21 deaths in the delivery room, 24 with gestational age ≥37 weeks, 10 with gestational age <23 weeks and 253 with gestational age 32-36^6/7^ weeks. The study group therefore included 627 preterm infants with a gestational age between 23^0/7^ and 31^6/7^ weeks, weighing between 500 and 1499 g, with no congenital malformations.

Table 1 presents the characteristics of the hospitals where the newborns included in the study were born: 13 (68%) met more than 60% of the criteria relating to hospital infrastructure according to the weighted score created for the classification and were classified as L1. Among the studied neonates, 76% were born in L1 hospitals. The number of neonatologists in the studied maternity hospitals was one per seven high risk neonates during the morning and one per ten during afternoon and night periods. For all working shifts, the median number of registered nurses per high risk neonatal bed was 1/10, with a minimum of 1/5 and a maximum of 1/21, without differences between L1 and L2 hospitals. The hospitals had, for all shifts, a median of one nurse technician per three neonatal intensive care beds (variation: 1/2 to 1/6), without differences between L1 and L2 hospitals.

**Table 1:** **Characteristics of the 19 maternity hospitals located in Northeast Brazil capitals and included in the study in 2007**

	**HOSPITALS**																		
	**1**	**2**	**3**	**4**	**5**	**6**	**7**	**8**	**9**	**10**	**11**	**12**	**13**	**14**	**15**	**16**	**17**	**18**	**19**
ICU exclusive for neonates	+	+	+	+	+	+	+	+	+	+	+	+	+	+	+	+	+	+	+
24-hour laboratory	+	+	+	+	+	+	+	+	+	+	+	+	+	+	+	+	+	+	+
Micromethods for blood exams	+	+	--	+	--	+	+	+	+	+	--	+	+	--	+	+	--	+	+
Blood gas analysis in the NICU	+	+	+	+	--	+	+	+	+	+	+	+	+	--	+	+	+	+	+
Bedside X-ray	+	+	+	+	--	+	+	+	+	+	+	+	+	--	+	+	+	+	+
Bedside ultrasonography	--	--	--	--	--	+	--	+	+	+	--	--	--	--	+	--	+	+	--
Bedside echocardiography	+	--	--	+	--	+	--	--	--	--	--	--	--	--	--	--	--	--	--
Parenteral nutrition available	+	+	+	+	+	+	+	+	+	+	+	+	+	+	+	+	+	+	+
Milk bank	+	+	--	+	--	+	+	+	+	+	+	+	+	+	+	+	+	+	+
Reference for high-risk gestation	--	+	--	+	+	+	+	+	--	+	+	+	+	+	+	+	+	+	+
Accredited as a safe maternity*	+	+	--	+	+	+	+	--	--	+	+	+	--	+	--	+	+	--	--
Pediatricians in the delivery room	+	+	--	+	+	+	+	+	+	+	+	+	+	+	+	+	--	+	+
Written guidelines for antenatal steroids	+	+	+	+	+	+	+	+	+	+	+	+	+	+	+	--	+	--	--
Bioethical committee	+	+	+	+	+	+	+	+	--	+	+	+	+	+	+	+	+	+	--
Hospital infection control committee	+	+	+	+	+	+	+	+	+	+	+	+	+	+	+	+	+	+	+
Maternal & neonatal deaths committee	+	+	--	+	+	+	+	+	+	+	+	+	+	+	+	+	+	+	--
Medical residence in Obstetrics	+	+	--	+	+	+	+	+	--	+	+	+	+	+	+	--	+	+	+
Medical residence in Pediatrics	+	+	+	--	--	+	+	+	--	+	--	+	+	+	+	--	+	+	+
Regular clinical staff meetings	--	--	--	+	+	+	+	+	--	+	--	+	+	+	+	+	+	--	+
Neonatal resuscitation training	--	+	+	+	+	+	+	+	+	+	--	+	+	+	+	+	+	+	+
Professional qualification training	--	+	--	+	+	+	--	--	+	+	--	+	+	+	+	+	+	--	+
Neonatal humanized care training	--	+	+	+	+	+	+	--	+	+	--	+	+	+	+	+	--	--	+
Weighted score (%)**	71.8	68.2	37.5	87.7	54.2	100	68.2	67.5	56.9	80.8	48.0	72.6	67.8	61.7	76.0	61.3	69.2	59.5	57.4
Hospital level	L1	L1	L2	L1	L2	L1	L1	L1	L2	L1	L2	L1	L1	L1	L1	L1	L1	L2	L2

*Accreditation attributed by the Brazilian Ministry of Health; **percentage of present variables for each hospital, according to the weighted score; Level 1 or 2 maternity according to the weighted score, being Level 1 those with better infra-structure.

**Figure 1:**
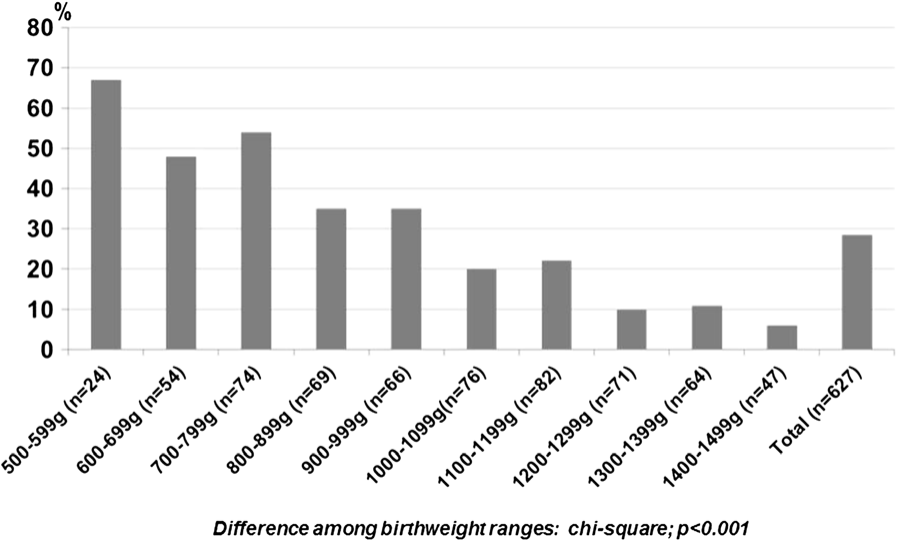
**Percentage of neonates that died up between 0–6 days after birth according to birth weight (grams).**

**Figure 2:**
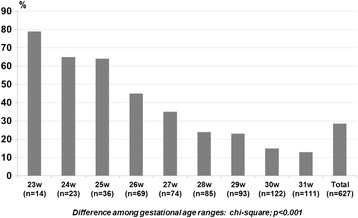
**Percentage of neonates that died up between 0–6 days after birth according to gestational ages (weeks).**

Among the 627 infants in the study, 179 (29%) died within the first 0–6 days hours of life. Of these, 59 (33%) died within the first 24 hours of life. The following distribution of deaths according to gestational age should be noted: the study included 216 patients between 23–27 weeks, of whom 38 (18%) died within 24 hours and 106 (49%) within 0–6 days after birth; 411 neonates were born between 28–31 weeks, of whom 21 (5%) died within 24 hours and 73 (18%) within 0–6 days after birth. The distribution of early neonatal deaths per 100 g strata of birth weight and per week of gestational age is shown in Figures 1 and 2, respectively. Early neonatal mortality was present in 26% (125/476) of patients born in L1 hospitals and 36% (54/151) of those born in L2 hospitals (p = 0.024). When hospitals were divided by number of intensive care beds, 25% (104/423) of neonates born in centers with more than 10 beds died in the first week of life and the same occurred for 37% (75/204) of those born in centers with 10 or less intensive care bed (p = 0.002). According to the Kaplan-Meier analysis, the probability of survival of the studied patients in the first week of life was 72%.

The distribution of maternal and neonatal demographic characteristics in relation to the presence of early neonatal death of the newborn can be seen in Table 2. Clinical complications in the newborns in relation to death in the first week of life are shown in Table 3. Variables related to procedures and interventions for neonatal care in relation to survival or death in the first week of life are shown in Table 4.

**Table 2:** **Maternal and neonatal characteristics according with the presence or absence of early death of preterm infants in the state capitals of Northeast Brazil (2007)**

	**Death <7 days (n = 179)**	**Survival ≥7 days (n = 448)**	**p**
Maternal age <20 years [n = 627]	56 (31%)	103 (23%)	0.021
Maternal age in years* [n = 627]	24 ± 7	25 ± 7	0.088
Schooling <8 years [n = 627]	90 (50%)	214 (48%)	0.316
Absence of prenatal care [n = 623]	40 (22%)	63 (14%)	0.009
Multiple gestation [n = 627]	34 (19%)	53 (12%)	0.015
Diabetes during gestation [n = 558]	3 (2%)	5 (1%)	0.396
Hypertension in gestation [n = 567]	52 (20%)	153 (37%)	<0.001
Peripartum infection [n = 558]	55 (36%)	144 (36%)	0.531
Antenatal steroids [any dose] [n = 596]	60 (36%)	230 (54%)	<0.001
Cesarean section [n = 623]	64 (36%)	222 (50%)	0.001
Birth weight <1000 g [n = 627]	129 (72%)	158 (35%)	<0.001
Birth weight in grams* [n = 627]	872 ± 229	1082 ± 242	<0.001
Gestational age <28 weeks [n = 627]	106 (59%)	110 (25%)	<0.001
Gestational age in weeks* [n = 627]	27.0 ± 2.3	28.8 ± 1.9	<0.001
Male [n = 627]	110 (62%)	208 (46%)	<0.001
1st minute Apgar score* [n = 604]	4 ± 2	6 ± 2	<0.001
5th minute Apgar score* [n = 607]	7 ± 2	8 ± 1	<0.001
1st minute Apgar <3 [n = 604]	46 (27%)	36 (8%)	<0.001
5th minute Apgar <7 [n = 607]	67 (39%)	48 (11%)	<0.001

*Variable expressed in mean ± standard deviation; brackets refers to the number of subjects of information available among the 627 studied infants.

**Table 3:** **Neonatal morbidity, according with the presence or absence of early death of preterm infants in the state capitals of Northeast Brazil (2007)**

	**Death <7 days (n = 179)**	**Survival ≥7 days (n = 448)**	**p**
PPV in the delivery room [n = 618]	138 (79%)	246 (56%)	<0.001
Advanced resuscitation [n = 596]	22 (13%)	15 (4%)	<0.001
Axillary temp. <36°C at admission [n = 569]	137 (92%)	333 (79%)	<0.001
Temperature at admission in °C* [n = 569]	35.2 ± 0.7	35.7 ± 0.7	<0.001
RDS [n = 619]	166 (95%)	372 (84%)	<0.001
Pneumothorax [n = 625]	8 (5%)	13 (3%)	0.218
PDA [n = 605]	10 (6%)	109 (25%)	<0.001
Early sepsis with positive BC (n = 614)	6 (4%)	28 (6%)	0.136
Any IVH [n with IVH/n with HUS (%)]	1/9 (11%)	104/308 (34%)	0.142
IVH 3–4 [n IVH 3-4/n with IVH (%)]	1/1 (100%)	21/104 (20%)	0.210

*Variable expressed in mean ± standard deviation; PPV: positive pressure ventilation; Advanced resuscitation: use of positive pressure ventilation plus chest compressions and/or medication; temp.: temperature; RDS: respiratory distress syndrome; PDA: persistent ductus arteriosus; BC: blood culture; IVH: intra ventricular hemorrhage; HUS: head ultrasound; brackets refer to the number refers to number of information available among the 627 studied infants.

**Table 4:** **Procedures and interventions for diagnostic and therapeutic neonatal care according with the presence or absence of early death of preterm infants in the state capitals of Northeast Brazil (2007)**

	**Death <7 days (n = 179)**	**Survival ≥7 days (n = 448)**	**p**
DR transport in incubator* [n = 600]	52 (31%)	210 (49%)	<0.001
Surfactant use [n = 627]	132 (74%)	269 (60%)	<0.001
Surfactant use ≤ 2 hours of life	90/132 (68%)	176/269 (65%)	<0.001
CPAP [n = 627]	59 (33%)	375 (84%)	<0.001
Mechanical ventilation [n = 627]	153 (86%)	316 (71%)	<0.001
Head ultrasound [n = 614]	9 (5%)	308 (70%)	<0.001
Umbilical catheter [n = 627]	149 (83%)	330 (74%)	<0.001
PICC [n = 627]	3 (2%)	131 (29%)	<0.001
Validated pain scale use [n = 600]	32 (19%)	122 (28%)	<0.001
Parenteral nutrition [n = 627]	72 (40%)	348 (78%)	<0.001
Parenteral Nutrition <24 hours of life	35/72 (49%)	159/348 (46%)	0.373
Hospital Level 1 [n = 627]	125 (70%)	351 (78%)	0.017

*Transport from delivery room to neonatal intensive care in a transport incubator; PICC: peripherally inserted central venous catheter; number in parenthesis refers to number of information available among the 627 studied infants.

The final Cox regression analysis model for the outcome of early neonatal death demonstrated its association with the following independent variables: absence of antenatal corticosteroid use (HR 1.56, 95% CI 1.09 to 2.23), multiple gestation (1.97, 1.29 to 3.00), male gender (2.01, 1.41 to 2.87), 5 minute Apgar <7 (2.98, 2.07 to 4.29), weight at birth <1000 g (2.58, 1.70 to 3.89), gestational age <28 weeks (2.03, 1.39 to 2.97), use of surfactant (1.64, 1.04 to 2.59), and lack of use of a pain scale (1.9, 1.24 to 2.9). The hypothermia variable (HR 1.31, 95% CI 0.88 to 1.96) remained in the final model because its withdrawal resulted in the loss of significance of other clinically important variables and risk/protection reversal, and therefore was considered a confounding factor.

## Discussion

The probability of survival in the first week of life for the infants studied here, between 23 and 31 weeks of gestational age and birth weight of 500–1499 g, was only 72%. This is lower than the rate found in 2004 and 2005 in the reference maternity units in the South and Southeast regions for those born between 23 and 33 weeks of gestational age (84%) [7]. In United States, between 2003 and 2007, hospital survival of newborn infants with a gestational age of 22–28 weeks, and therefore more immature than those analyzed in this study, was 72% [8]. A multicenter study of European countries in 2003, in turn, found a hospital survival rate of 89.5% for infants between 22–31 weeks of gestational age [9].

Of the 627 infants studied, 59 (9.4%) died within the first 24 hours. In a 2004 Brazilian Neonatal Research Network study of university public maternity units in southeastern Brazil, of the 560 patients with a birth weight between 400 and 1499 g, excluding deaths in the delivery room, 25 (4.5%) died within the first 24 hours [7]. In a cohort study conducted between 1997 and 2004 in the United States on neonates with birth weights between 500–1499 g, among the 91,578 studied, 4,579 (5%) died within the first 24 hours [10]. The earlier the death of the newborn, the more it is connected to social and economic determinants related to the quality of the mother's health care [11].

Of the 19 hospitals selected for this study, neonatal survival rates were significantly higher in L1 hospitals with more than 10 neonatal intensive care beds. Other studies observed that mortality of preterm infants is lowest for deliveries that occur in hospitals with NICUs that have both a high level of care and a high volume of such patients [12]. Technological resources, such as ultrasound and echocardiography at the bedside, and clinical meetings that provided the ability to reflect on the medical practices performed and learn from possible mistakes and omissions were absent from most institutions classified as L2. That is, although all analyzed hospitals were public, some invested more in diagnostic resources for premature patients, who are dependent on technology for survival, and some invested more in human resource training, which is fundamental to implementing the technological resources for this extremely vulnerable population. It is noteworthy, however, that some institutions considered as L1 did not have regular clinical staff meetings, did not require neonatal resuscitation training, professional qualification training or neonatal humanized care training, which points out that even for the better hospitals included in this study, investments in continuous education of health professionals should be done to improve neonatal care.

The independent risk factors associated with early neonatal death observed in this study included some commonly reported variables such as the absence of antenatal corticosteroid use [13], multiple gestation [14-17], male gender [14,15,18], five minute Apgar <7 [7,19,20], birth weight <1000 g [14,15,21] and gestational age <28 weeks [7,14,16,22,23]. The contribution of these variables to early neonatal deaths indicates that the biological characteristics related to the vulnerability of the preterm infant (birth weight, gestational age, gender and twinning) and vitality at birth (5 minute Apgar score <7), and characteristics reflecting the care of pregnant women in the peripartum period and the training of pediatric staff who attend the newborn in resuscitation and life support, are key determinants of the success of neonatal care in the first days of life. In the present study, the gestational age at which survival beyond 6th day of life exceeded 50% was 26 weeks, indicating that it is necessary to invest in perinatal health in the analyzed region to rectify the inequality in viability for premature infants born in this area.

Meta-analyses show that the use of antenatal corticosteroids has a protective effect against neonatal mortality in premature infants born at 24–34 weeks of gestational age [14,24]. Despite the universal recommendation for antenatal corticosteroids in gestation at risk of preterm delivery before 34 weeks of gestation, they were used in only 49% of cases in this study. These data exceed the 22% use of antenatal corticosteroids obtained from a population-based cohort of 774 VLBW infants born in Fortaleza, in the northeastern region, between the years 2002–2003 [25], but are below the 25th percentile (P) reported for the use of corticosteroids in live births without malformations in the Brazilian Neonatal Research Network (P50: 65%, P25-75: 51-72% in 2008) [26]. In the Vermont Oxford Network, between 1998–2006, an increase in the use of antenatal corticosteroids from 77% to 85% was identified when evaluating 4,065 VLBW newborns [27]. In United States, a study of 9,575 infants with a gestational age between 22 and 28 weeks and weighing 401 to 1500 g found that antenatal corticosteroids were used in 83% of cases between 2003 and 2007 [8]. In Northeast Brazil, the movement of at risk pregnant women occurs from the interior to the capital cities in a pilgrimage through hospital emergency rooms, increasing obstetric risk and allowing for a series of missed opportunities for the administration of medication [28].

The increased administration of antenatal corticosteroids in the 1990s and the use of surfactant for respiratory distress syndrome have been the perinatal treatments with the greatest impact on early neonatal mortality [13,29,30]. Surprisingly, the use of surfactant in this study was associated with the risk of early neonatal death. This relatively expensive resource was available in the studied units: among the 627 neonates, 401 (64%) received surfactant after birth and 266 (66%) of them in the first two hours of life. Also, the preparations used in these patients were those available internationally, namely Cursosurf® and Survanta®. That is, despite the availability of the medication and its effectiveness in reducing neonatal mortality in randomized controlled trials [29], the surfactant was associated with a 60% increase in the risk of death in this study. Newborns whose clinical condition is more severe require more physical infrastructure, equipment and human resources for their survival, along with the careful integration of these features. The use of surfactant seems to indicate that the newborn had to be intubated and receive mechanical ventilation, involving a complexity of care that existing structures in the evaluated maternity units were not able to offer.

The use of analgesia in newborn care in worldwide neonatal care units is still controversial and irregular [31]. In the present study, the group of newborns for whom the professional team did not apply a validated pain scale during admission had twice the risk of death in the early neonatal period. The lack of pain assessment in critically ill premature infants does not have a physiopathological relationship with progression to death, so the presence of this variable in the final model appears to be due to its significance as a marker of the organization of neonatal care. The low use of a pain scale for newborns in the Northeast Brazil units studied here reflects a failure in the care process.

Finally, hypothermia upon admission to the NICU was an important adjustment variable in the explanatory model of early neonatal death. Laptook et al. [32], studying 5,277 VLBW preterm infants at 15 U.S. centers in 2002–2003, found that in-hospital mortality was inversely proportional to temperature at admission. In a Brazilian Neonatal Research Network study, a prospective cohort of 1,764 patients between 22–33 weeks gestational age, without malformations, born between 2010 and 2012 was analyzed. Hypothermia upon admission to the NICU was diagnosed in 51% of newborns and increased the chance of early neonatal death by 1.64 times (95% CI 1.03 to 2.61) [33]. It is therefore essential to plan feasible strategies for thermal protection of the newborn and to reduce the incidence of hypothermia on admission to the NICU, protecting the patient from the complex web of factors related to poor quality of perinatal care, the outcome of which is death.

## Conclusions

It is important to emphasize that the use of secondary data means that there are limitations and difficulties inherent to the methodology itself. Also, the fact that data were collected in 2007 brings a question regarding the validity of the results nowadays. In this regard, despite improvements in health indicators of the Northeast Region of Brazil, early neonatal mortality rate in 2012 was still 20% of the live births with gestational age 22–31 weeks [34] and variables associated with these deaths are largely understudied. Finally, we did not analyze variables associated with early neonatal death in each birthweight or gestational age stratum because the study was not designed and powered to perform this analysis. Despite these limitations, this is the first study with prospective data collection from reference maternity units in Northeast Brazil and it provides a picture of care at birth for preterm infants with very low weight, which contributes substantially to infant and child mortality and influences the human development index in this region.

In conclusion, beyond biological variables, factors related to the quality of perinatal care were associated with the high chance of early death of preterm infants born in reference hospitals of Northeast Brazil.

### Ethics approval

The Institutional review Boards from the Federal University of São Paulo and Federal University of Ceará. The Clinical Board of each participating institution approved the study protocol.

## Consent

The study was done as a qualitivieve initiative of the Ministry of Health of Brazil that funded RENOSPE (Rede Norte-Nordeste de Saúde Perinatal) . The collection of data was approved by the Clinical Board of each hospital and by the IRB of the main institution related to RENOSPE withouth the need of parental informed consent (Maternidade Escola Assis Chateaubriant and Federal University of Ceará). The IRB of the main institution for the present study (Federal University of São Paulo) approved the study with the data collected from RENOSPE. As the study relates to the use of a database without any intervention, the Federal University of São Paulo approved the use of the data under confidentiality os patients' identity.
